# Harvard is Spending, Education Needs Mending

**DOI:** 10.1100/tsw.2001.3

**Published:** 2001-01-19

## Abstract

Nature leads with happy news about a spending spree at Harvard University. Yet another study, this time from a student perspective, argues that graduate education in the U.S. is deeply flawed, reports Science in this week's lead story.

The administration of Harvard University is planning on dumping hundreds of millions of dollars into a new natural history museum, more faculty, and extra construction, reports *Nature* in this week's lead story. The new museum will house several diverse scholarly collections, including the university's popular Glass Flowers collection of more than 4,000 botanical models, and free up space for offices and laboratories, reports *Nature*. The generous windfall, which could reach a shocking $700 million, comes courtesy of Harvard's ballooning endowment, says *Nature*. Now valued at $19.2 billion, the endowment grew by 32% in the year ending last June. *Nature* quotes Jeremy Knowles, dean of the faculty of arts and sciences, who explains "Harvard plans to grasp the opportunity to improve the quality of the undergraduate experience and the lives of graduate students and faculty." With $700 million to spend, TWINS can't imagine that Harvard could fail to achieve this modest goal. The article did not mention what effect the several-month-long U.S. stock market slide might have had on the value of Harvard's endowment or their future spending plans. *Science* did not cover this story.

*Science* leads with a story about the "three-way mismatch between student goals, their training, and their actual careers" in U.S. graduate education. According to a new study funded by the Pew Charitable Trust, grad students generally believe that graduate school prepares them to be faculty at research universities. Although students do learn top-flight research skills, concludes the report, graduate schools do not teach other skills, such as teaching and mentoring, necessary for a career as a faculty member, reports *Science*. All of which is beside the point, say the report authors, because only a small minority of graduate students ever realizes their dream of becoming full-time faculty at research universities. *Science* states that the report found "almost half of U.S. faculty members are part-timers, nearly one-third work at 2-year colleges, and only one-quarter of full-time faculty members work at research universities." Condensing it even further, the figures imply that only one in eight faculty members works full-time in a research university.

The report also offers some recommendations to improve the situation, says *Science*. The authors suggest "providing more relevant information for students about their training and what happens to graduates, better mentoring by faculty, more emphasis on teaching, more courses outside one's field, and more discussion of the problem at professional meetings." None of this is new, acknowledge the authors, so why does the system persist in training uninformed students poorly for jobs that don't exist? "The incredible conservatism of higher education" and the fact that professors who have thrived under the current system have little incentive to change it, says report co-author Chris Golde in the *Science* article. But there was good news: 91% of graduate students "like their advisor." *Nature* did not cover this story.
